# Physical and Thermal Properties Analysis of Hematite for Thermal Heat Storage

**DOI:** 10.3390/ma15134648

**Published:** 2022-07-01

**Authors:** Andreia Santos, Fernando Almeida, Fernando Rocha

**Affiliations:** GEOBIOTEC, Geosciences Department (DGeo), University of Aveiro, 3810-193 Aveiro, Portugal; fernandoalmeida@ua.pt (F.A.); tavares.rocha@ua.pt (F.R.)

**Keywords:** thermal energy storage, iron oxidized, Moncorvo iron deposit, sensible heat, thermal conductivity, specific heat

## Abstract

Energy sustainability represents an important research topic for aiding decreasing energy dependence and slowing down climate changes. In this context, solutions using thermal energy storage through rock start to emerge, due to its natural benefits, when compared to more polluting alternatives. To understand whether a rock material can be considered a good thermal energy storage material for such solutions, it is necessary to evaluate the physical, chemical and thermal properties of such materials. Therefore, it becomes essential to understand how heat propagates in the rock and how voids influence the thermal properties. To achieve these goals, hematite ore from Moncorvo, Northeastern Portugal was used, in particular, to study the effect of grain size on thermal properties for three different sized lots. Chemical and physical changes between heated and unheated lots were detected using X-ray diffraction and particle size, as well as X-ray fluorescence analysis. Regarding thermal properties, a hot wire method approach was used with seven thermocouples. Additionally, a thermal inversion model to simulate the heat exchanges was also proposed, allowing changing the properties of the constituents, to fit the theoretical and experimental temperature curve. Furthermore, the model reveals how heat propagates inside the reservoir filled with hematite ore.

## 1. Introduction

In recent years, the challenges associated with energy sustainability have been key aspects of the research agenda, and with it, several ideas/projects have started to emerge, with the aim of producing or managing energy, focusing on decrease energy dependence, while also helping to slow down climate changes [1].

In the field of geology, there are several approaches to this challenges, such as the use of geothermal energy and thermal energy storage in aquifers, ponds and rock beds [2,3,4,5,6]. These strategies have been explored in some developed countries as alternatives to polluting energies, e.g., in Turkey, a two-hole aquifer was used to acclimatize an area of 1400 m${}^{2}$ in a supermarket in Mersin (near the Mediterranean coast) [7]. These types of approaches can be used for long or short periods of time, i.e., heat can be stored during day to night and, more evolved, stored during the summer and used in winter [8,9,10,11]. Other strategies are the use of geothermal energy, especially in volcanic areas where the geothermal gradient is high, prompting the use of this type of renewable energy. Examples include the Philippines, where 21% of electricity comes from geothermal steam. The same occurs in El Salvador (20%), Nicaragua (17%), Costa Rica (10%), and others [12]. Another technique for storing energy in rock consists in using salt rock storage with compressed air, which has been the target of great investment in China [13,14].

The use of rock materials capable of storing sensible heat offers great advantages, such as lower price, non-toxic, non-flammable, and lower environmental impact. It can be a secondary material for exploration, making better use of the resources already used. In short, it has a lower environmental impact because it avoids the depletion of natural resources, which can be stored underground (occupying less space on the surface) [15,16,17].

To choose a rocky material that allows a better effectiveness in storage of sensitive energy, it is necessary to take into account some parameters including physical, chemical and thermal properties. Among many existing characteristics, it is important to consider the following: material porosity, the structure of the mineral/rock, density, stability, thermal conductivity, sensible heat, as well as the hardness of the rocky material. Furthermore, it must have some resistance and not suffer changes during the heating and cooling cycle in order to avoid damage to the storage system [18,19,20]. However, these properties depend on how you want to propagate the heat in the rock material [21].

In summary, a good heat-sensitive rock material must include special characteristics, such as being stable during heating and cooling cycles, having a high specific heat value to be able to absorb more heat, and finally, having a low thermal conductivity, thus preventing heat from being transmitted to the surroundings (so that there is less energy loss). However, it is also important that the material can yield the absorbed heat when required, and also the balance of thermal conductivity must be taken into account.

Therefore, knowledge of the physical, chemical and thermal properties of rock materials become an important and essential subject [22,23,24,25]. To achieve a good characterization of thermal properties, it is necessary to know the temperature distribution and the thermal gradient of the material [26,27]. In this context, the determination of energy distribution and heat flow is a problem that interests many areas of engineering and science, from heat exchanger projects necessary for their sizing (heat pumps, boilers), nuclear reactors, space technology, insulation and geothermal (terrestrial or space) [28,29].

In this paper, the physical, chemical and thermal properties of the hematite ore from Moncorvo (Cabeço da Mua) are characterized to assess the suitability of this geomaterial for thermal energy storage. For this purpose, we divided the rock material into different batches and checked whether there were changes relative to the heated and unheated batches in the following properties: chemical, physical, in the structural order, phase composition and microstructure of the minerals. We used the following methods: X-ray diffraction (XRD) and X-ray fluorescence (XRF) analysis, particle size analysis and, to assess the thermal properties, an approach to the hot wire method. Finally, to understand the heat flows in the hematite ore, lots with different granulometries were tested, which is more efficient for the process.

## 2. Study Area

**Figure 1:**
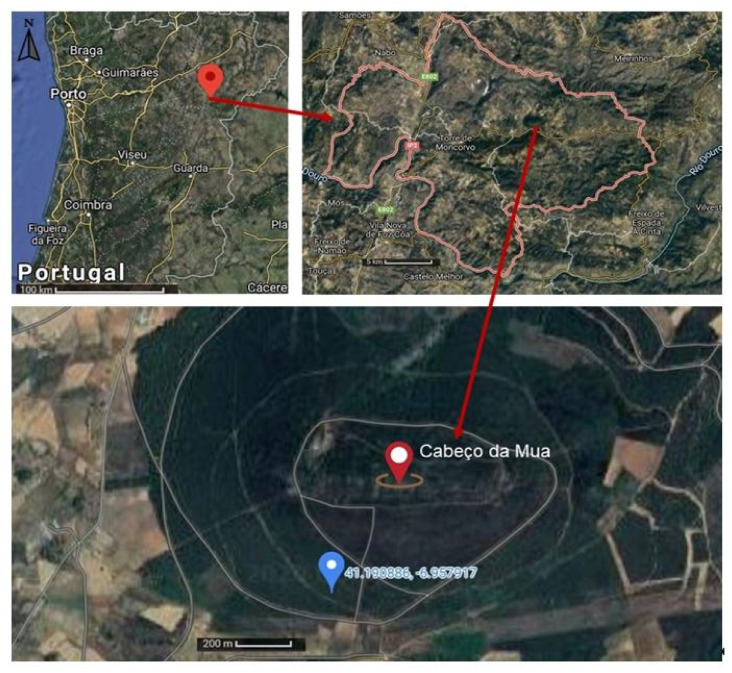
Location of the Cabeço da Mua hematite ore sampling area, Moncorvo. Images obtained from Google Maps.

In this section, we present the literature on regional geology and characterize the rock material used in the experiments. The rock material used in the experiments was the hematite ore collected in Cabeço da Mua, Moncorvo, situated at Northeast Portugal as shown in Figure 1. The Cabeço da Mua is located in the Moncorvo synclinium, in the Central Iberian zone, resulting from a long process of deformation during the Variscan Orogeny.

The iron deposit of Moncorvo formed during the Ordovician period as carbonates or hydrated oxides in the marine environment. During the Variscan orogeny (245 million years ago), they suffered regional metamorphism causing the transformation of primary iron minerals into magnetite occurred. Then, hydrothermal metamorphism occurred that originated martitization and the genesis of specularite (hematite form) [30].

**Figure 2:**
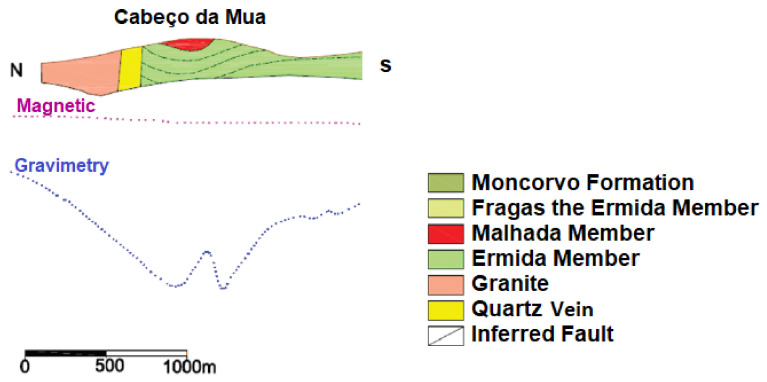
Longitudinal section (N−S) on Moncorvo’s synchlorium with overlapping interpretation of geophysical data. Adapted from [31].

The Moncorvo Mineralized Complex, to which the Cabeço da Mua and the Carvalhosa belong, has high textural, mineralogical and facies variability. At the structural level, it contains three members: Fraga da Ermida, Malhada and Ermida. Considering Figure 2, we can observe in the north of the Cabeço da Mua a quartz lode and an intrusion of granites. The Cabeço da Mua and the Carvalhosa belong to a synclinal, where the member Malhada contains the minerals of iron (hematite and magnetite) with lateral variations of quartzite and the Ermida member contains quartzites [31].

In particular, the iron deposit of Cabeço da Mua is estimated to have 73.42 Mt with 42.7% iron grade containing mainly hematite but there are several iron minerals, such as magnetite, goethite (in different proportions) and secondary minerals, such as quartz and muscovite. The main phosphate minerals are lazulite-scorzalite, apatite and rockbridgeite. Limonite, biotite, sericite and chlorite are also present in lower quantities [31]. The thickness of the iron ore is very variable. Cabeço da Mua represents a layer with a maximum thickness of almost 90 m, where the mineralized sequence is practically continuous. The ore is predominantly hematite, although it contains other minerals, such as magnetite, muscovite, phosphate and quartz interbedded in the sequence. The iron mineral occurs associated with the upper quartzite of the Marão Formation, Malhada member [32].

## 3. Methods and Materials

As thermal properties are conditioned by the intrinsic characteristics of the material, this section describes the methods used to characterize the samples submitted to the heating and cooling experiments at mineralogical, morphological and chemical level. Several laboratory experiments were performed, such as mineralogical analysis by XRD, chemical analysis by XRF. To characterize the physical properties, a granulometric analysis was performed and the density and porosity of each lot was calculated, because the porosity has an influence on how the heat flow propagates through the material, since the thermal conductivity of air (empty spaces) is very low.

Although, to ensure that there were no changes in the samples during the heating and cooling cycles, the same experiments were performed on samples that were not heated and compared with those that were heated, because chemical or mechanical changes (which can happen during the heating and cooling cycles) can bring great problems for the stability and efficiency of thermal energy storage.

Mineralogical analyses were carried out by XRD, using a Panalytical X’Pert-Pro MPD, CuK α${}_{1}$${}_{,}$${}_{2}$ (λ = 1.5405 Å) radiation; the identification and semi-quantification of the different mineral phases followed the criteria recommended by [33,34,35] and the Joint Committee for Powder Diffraction Standards. Determination of chemical composition was assessed by XRF using a Panalytical Axios PW4400/40 for major and trace elements; lost on ignition (LOI) was also determined (heated at 1000 °C for 2 h). For XRD and XRF analyses, only the total distribution lot (TD) was used because all lots were chemically equal since the coarse fragmentation carried out did not reach hematite grain liberation, assuming that the natural release of hematite was not promoted.

**Figure 3:**
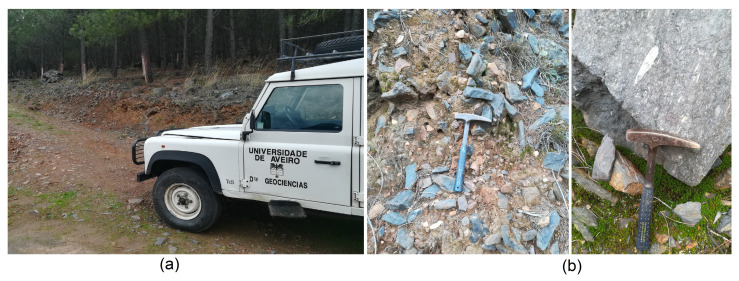
Hematite ore sample extraction area, located on the slopes of Cabeço de Mua, Moncorvo: (**a**) sample collection site along a 100 m stretch; (**b**) hematite ore in situ.

A considerable amount of rock fragments, about 0.5 m${}^{3}$ of rock, was collected at the surface of the coluvium in a space of one hundred meters to be representative of the materials that emerge in the area. In Figure 3, it is possible to verify the extraction site located in one of the slopes of the Cabeço da Mua. The dispersed rocks were in contact with the chemical and mechanical weathering agents (Figure 3b).

**Figure 4:**
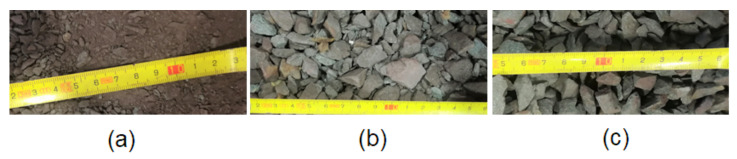
Different lots of hematite ore: (**a**) undersize—US; (**b**) total distribution—TD; (**c**) oversize—OS.

The collected hematite ore was studied with minimal intervention and was submitted to a process of mechanical fragmentation, first with a hammer and then using a jaw mill. In order to evaluate the importance of the granulometry in heat storage, the hematite ore was passed through a 4.75 mm sieve, from where three chemically equal lots resulted, but with different granulometries (Figure 4):

- Undersize (US): granulometry inferior to 4.75 mm;
- Total distribution (TD): total granulometry of the sample;
- Oversize (OS): granulometry higher than 4.75 mm.

For the determination of the physical–chemical parameters, the hematite ore was subjected to heating/cooling cycles (AQ) and samples of control were not subject to heating (NAQ). Next, a grain size analysis was performed on the three unheated lots. Plus, another three lots were used in the heating experiments. This helped us to understand the particle size difference between the lots, OS, US, and TD, and whether there was a change in particle size after the heating/cooling cycles. For this, dry sieving was used, a method of easy practice, where it is necessary to use a set of sieves with different mesh diameters; in this case, nine sieves (12 mm; 9.5 mm; 4 mm; 2 mm; 1 mm; 0.5 mm; 0.25 mm; 0.125 mm, and 0.063 mm) and a mechanical vibrator.

Additionally, to calculate the density and porosity of the rock material, it was necessary to weigh a sample from each lot that was heated with a cylinder with 0.23 m in height and 0.11 m in diameter, i.e., with a volume of 0.002186 m${}^{3}$. The porosity (Ø) was calculated using the following Equation (1), where the density of the aggregate ($\rho_{a}$) was obtained in the previous step, and the average density of the hematite ore ( $\rho_{s}$) was calculated using the Jolly scale and 3970 kg/m${}^{3}$ [36].

(1) $$Ø=1-(\rho_{a}/\rho_{s})$$

However, during the mineralogical and chemical analysis only the heated and unheated TD samples were used because there were no chemical and mineralogical differences between the lots, since they all came from the same sample and there was only a grain size separation among them.

Based on these analyses, it was possible to characterize the samples and verify if there were changes due to the heating and cooling cycles. Furthermore, to determine the thermal properties of the material, a laboratory scale reservoir was built (with 0.23 m in height and 0.11 m in diameter) filled with granulated hematite ore. In this context, and to ensure greater precision, all the necessary steps for manufacturing were considered, ranging from the cylindrical reservoir, to the insulation and the heat source. In addition, using the LabView software that controls an acquisition board (DAQ) with seven thermocouples, we were able to obtain a file with the temperature in the seven thermocouples over time, thus recording the heating and cooling periods. This file was essential to implement a numerical model made in Matlab software, for calculating the thermal and physical properties of hematite.

To characterize the samples thermal properties, it was necessary to build a thermally insulated reservoir with several thermocouples and a heat source in the center. This method is based on the well-known hot wire method, which features an infinite wire that generates heat [37,38,39]. To calculate the thermal conductivity and the specific heat of the rock material, it was necessary to use a routine in Matlab software. The routine consisted in taking the thermal properties through the Fourier equations, with the thermal and physical properties of each element (reservoir, hematite, heat source). The program computes the curve of the reservoir temperatures (theoretical curve) and compares it with the curve of the experimental temperatures (experimental curve). Through inversion/optimization, the thermal and physical properties are changed to overlap the theoretical curve to the experimental one. Thus, obtaining the thermal and physical properties of the hematite ore.

## 4. Results and Discussion

**Figure 5:**
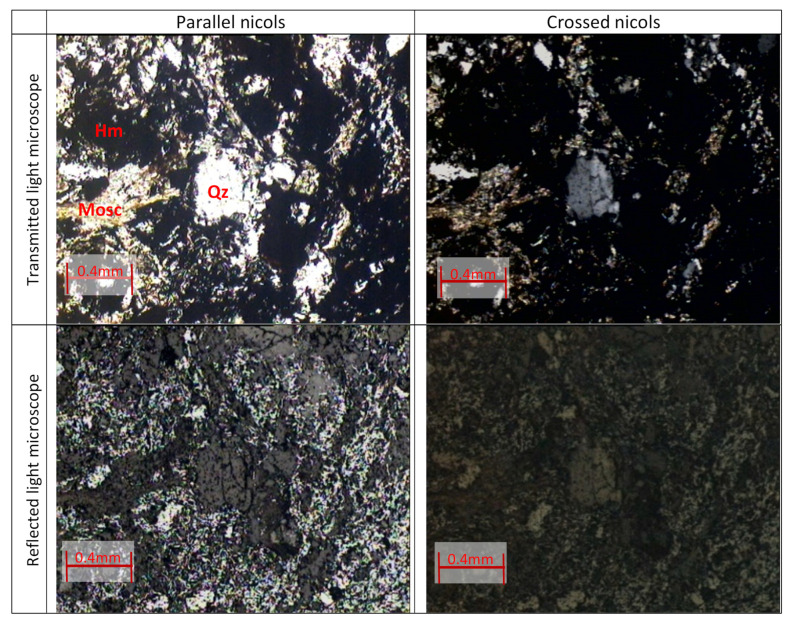
Observation of the polished surface under the microscope with transmitted and reflected light, in parallel and crossed niches. A matrix of hematite (small-grained) is observed, containing quartz and muscovite. The amount of hematite is about twice that of quartz.

Figure 5 shows four images of the polished surface under a microscope with transmitted and reflected light, in parallel and crossed niches. The mineralogical constitution is composed of quartz showing granular texture, muscovite that gives the rock the lepidoblastic texture, and fine-grained hematite constituting the matrix that becomes opaque in transmitted light microscope and reflective in the microscope reflected light. Thus, the overall texture of the rock is of the granololepidoblastic type and the amount of hematite is about twice as much as quartz.

**Figure 6:**
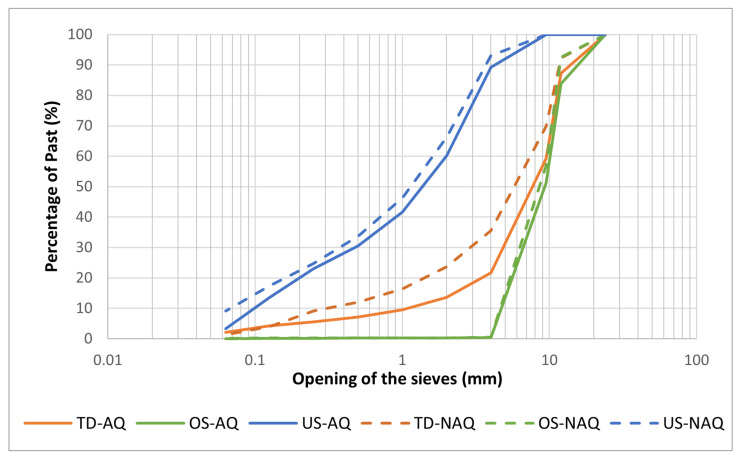
Percentage curves of passes according to the opening of the sieves of all the samples.

Regarding the particle size analysis through dry sieving, the OS sample is constituted with grains mostly with a size superior to 9.5 mm. In contrast, on the US sample, 50% of the grains are inferior to 1 mm. Since the TD sample was the first to undergo calibration, forming the two other lots, its grains are more heterogeneous. Comparing the heated and unheated samples, there were no major changes in the OS and US lots. However, in the TD lot there is a slight difference, a higher number of grains with smaller size. This may have occurred during the heating/cooling cycles or by the handling of the sample itself. Figure 6 shows the granulometry curves of the three lots and the curves of the heated and unheated lots.

Aggregate density results were for OS 1925.96 kg/m${}^{3}$, for TD 2170.82 kg/m${}^{3}$ and for US 2262.688 kg/m${}^{3}$. After applying Equation (1), the porosity result was 0.515 for the OS lot, 0.453 for the TD and 0.430 for the US lot.

**Figure 7:**
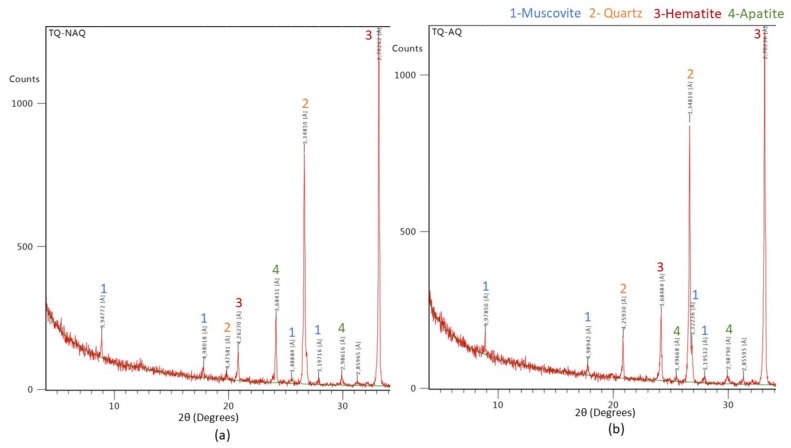
XRD powder diffraction analysis with identified peaks: (**a**) TQ sample not subjected to heating/cooling cycles; (**b**) TQ sample subjected to heating/cooling cycles.

The results of mineralogical analysis by XRD show that the peaks of the TD sample with and without heating are similar, emphasizing the argument that there were no mineralogical changes (Figure 7). Comparing the shape and size of the peaks, we can assume that there were no changes in the structure of the minerals. This conclusion is very important, as it ensures that the hematite ore is stable and does not change in any way during the heating/cooling cycles. Regarding the peaks with the highest count, hematite and quartz are identified, being in accordance with polished thin section observations and with the literature consulted. As accessory minerals, muscovite and apatite were also identified, which also meets the information found in the literature. Apatite is a mineral that contains phosphorus, which is penalizing in iron metallurgy. In the semi-quantification of the identified minerals, hematite has a higher percentage in both lots, about 68%, followed by quartz with about 29% and finally apatite and muscovite with about 0.5% and 2%, respectively.

Table 1 shows the minerals found in the lots samples, as well as their chemical composition, description and semi-quantification.

Chemical analysis by XRF shows that the oxides with the highest percentage are Fe${}_{2}$O${}_{3}$ (66.5%) and SiO${}_{2}$ (23.5%), which is compatible with the analysis of diffractograms that indicate that the most abundant minerals are hematite and quartz. Next, we have Al${}_{2}$O${}_{3}$ (6%) and K${}_{2}$O (1%), related to muscovite. Finally, P${}_{2}$O${}_{5}$ (0.6%) and CaO (0.124%) are related to calcium phosphate as apatite. However, there are other relevant oxides that do not fit the minerals found in the XRD analysis, such as Na${}_{2}$O and TiO${}_{2}$; the latter may be inserted in hematite, forming ilmenite-hematite or ilmenite (Table 2).

Comparing the XRD and XRF results (Table 2), all iron oxide is hematite. However, XRD values for hematite and quartz are affected by the presence of amorphous, non-silicate materials, such as alumina hydroxides, that do not appear clearly in diffractograms. This also explains the high XRF values of aluminum oxide, comparing with a muscovite amount of 1% in XRD semi-quantification.

In the first step, the results of physical and thermal properties were obtained for the three constituents, for a total of nine parameters (specific heat, thermal conductivity and density). This was done by testing the three lots with different particle sizes for varying heating and cooling times (between 1 h and 12 h).

**Figure 8:**
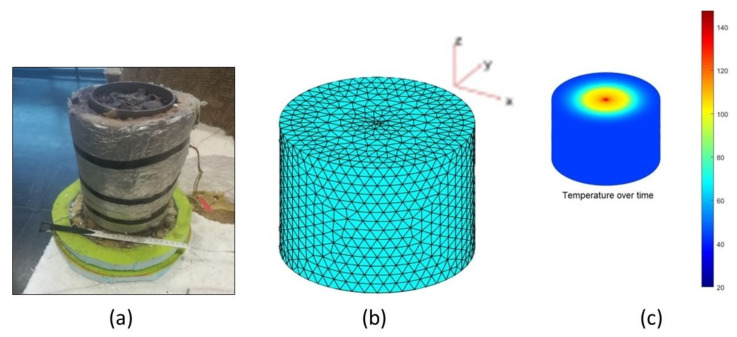
Reservoir overview: (**a**) experimental reservoir filled with the hematite ore; (**b**) theoretical model developed in Matlab with the inversion points; (**c**) temperature distribution in the reservoir over time.

In Figure 8a, it is possible to observe the reservoir built in the laboratory, filled with hematite and with the glass wool insulation. In Figure 8b, it can be found a model of the reservoir obtained in Matlab, where the inversions are performed in each node of the blue triangles. Finally, Figure 8c refers to the distribution of temperatures over time inside the reservoir.

**Figure 9:**
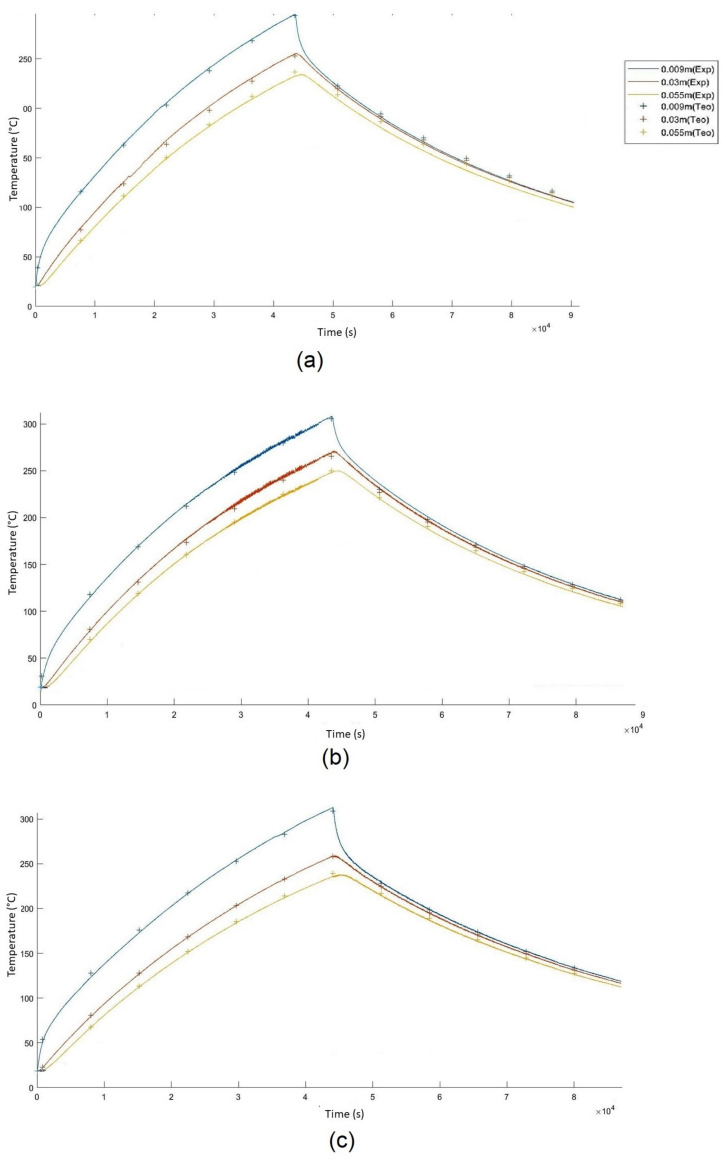
Theoretical and experimental curves for a heating and cooling cycle. The crosses represent the temperatures returned by the software and the line the actual temperatures: (**a**) temperature graph for lot TQ; (**b**) temperature graph for lot OS; (**c**) temperature graph for lot US.

Figure 9 show the graphs obtained through Matlab of the heating/cooling cycles. The solid lines represent the experimental temperature curve, and the crosses represent the theoretical temperature curve, returned by the theoretical model, with the best optimization (RMS). The experiments were performed with a combined duration of 12 h heating and 12 h cooling. The thermocouples were placed inside the reservoir with the hematite ore at the following distances: 0.009 m; 0.03 m; and 0.055 m. Inside the insulation, they were placed in its center at the following distances of 0.08 m, 0.11 m, and 0.14 m. Comparing the heating curves of the three lots, you can see that the differences are subtle. All the curves have the same shape, which is predictable because the material is the same. Furthermore, this was also expected, given that this is the normal shape for this type of sensible heat storage. Additionally, Figure 9 proves that the TD material absorbs more heat because although the energy delivered is the same in all experiments, the thermocouples in the TD do not reach the 300 °C temperature as in the other lots, which is caused by the hematite grains absorbing more energy. However, the TD lot also loses temperature faster because it has a higher conductivity than the other lots (Figure 9a). It is the US lot that is able to store heat longer (Figure 9c).

In order to optimize the inversion process, some parameters were fixed. The thermal and physical properties of the insulation and the heat source were tested in several heating and cooling cycles, and the model returned approximate values. In addition, the values obtained for the insulation were identical to the ones presented in the technical tables. Finally, the density value of the 3 aggregates was also fixed, using the values obtained in the characterization of the hematite ore, described at the beginning of the article.

In Table 3, the thermal properties and density values of all the lots are presented, as well as the RMS value for each optimization.

When analyzing the values of each lot, it was verified that the values obtained through the model for the specific heat were low, compared to the previously tested hematite ore [40], which was of the order of 756.76 J/kgK. This result leads us to conclude that the value obtained is accounting for the effect of the voids and not the value of the compact rock. It is evident that the voids are important and influence the final value of the specific heat. To overcome this issue, it may be permissible to further dilute the specific heat into the cylindrical volume of the reservoir, since the heat equations are written for continuous media. Since the heat flux percolates through the solid phase of the aggregate, it is only continuous throughout the perimeter if we normalize the specific heat to the fraction of solids ($c_{solid}$), diluting it into the voids to obtain the specific heat of the aggregate ($c_{aggregate}$) according to Equation (2), where (Ø) is the porosity.

(2) $$\left(c_{aggregate}\right)=\left(1-Ø\right)*c_{solid}$$

Transforming $c_{aggregate}$ to $c_{solid}$ produced results closer to 756 J/kgK obtained earlier for $c_{solid}$ (Table 4). Therefore, we verify the importance that porosity has on thermal properties due to the low specific heat of air that does not store heat in an effective way but leads the modeling in aggregates to consider this aspect.

Regarding the parameters obtained for the specific heat in the modeling, it can be seen that the TD lot, where the particle size distribution is wider, is the one presenting a higher specific heat. In other words, the TD is the most suitable lot for storing specific heat in rock.

This way of storing thermal energy in rock can be used in domestic and/or industrial situations, with just the dimension of the cylindrical reservoir for this purpose. This solution is capable of moving excess energy, produced by a wind or solar panel, to a height where it is not being produced. This maximizes the renewable energy used and gives another use for a material that can be economically profitable to extract again.

## 5. Conclusions and Future Work

Aiming to build a reservoir for storing thermal energy with high sustainability, the Moncorvo hematite ore aggregate chemical, thermal and physical properties were assessed. The objective was to study the effect of grain size on thermal properties using three different size lots. In this context, several experiments were carried out: petrology, particle size, XRD and XRF. They allowed to understand the characteristics of the samples, as well as to characterize them accordingly. Based on the results obtained, we were able to compare the samples that were subjected to heating/cooling cycles with the ones that were not.

The lots tested in the experiments were undersize (US), total distribution (TD), and oversize (OS). Through particle size analysis, we can conclude that the US lot has 50% of the particle sizes below 1 mm, the OS lot has particle sizes above 9.5 mm, and the TD lot has heterogeneous particle sizes between 9.5 mm and 1 mm. The aggregate density is high, as well as the porosity: TD has 2171 kg/m${}^{3}$ density and 0.45 porosity, US has 2263 kg/m${}^{3}$ density and 0.43 porosity, and, finally, OS has 1926 kg/m${}^{3}$ density and 0.52 porosity.

Using XRD and XRF methods revealed the chemical and mineralogical properties of the hematite ore, which contains a large percentage of iron oxide (hematite, 66%), quartz (30%) and the remaining 4% is muscovite and apatite. No difference was observed between the lots that were subjected to heating/cooling cycles and the lots that were not heated, in terms of grain size, structural order and phase composition. This result reinforces the confidence in using this material since it is stable for use in the reservoir.

The results of the thermal properties obtained through the hot wire method approach and inversion model showed that the thermal conductivity of the hematite grained ore is low 0.3 W/mK for the TD and OS lots, and 0.25 W/mK for the US lots, which allows energy to be stored for longer periods. The specific heat of the solid is around 618 J/kgK, 668 J/kgK for OS and 554 J/kgK for US, which allows us to infer that the grains of hematite ore are able to absorb more energy, thus showing that it is a good heat storage material, as long as there is a greater number of connections between the grains. Furthermore, the larger the grain, the more heat it can store and the faster the heat flow will be in the aggregate.

Hence, the lot that meets all the desired characteristics for energy storage is TD since it has the best thermal properties and suitable fragmentation. Moreover, the energy consumption in fragmentation is low because it is not necessary to reduce to finer grains. Additionally, the fines are not rejected from the process.

Future work will include testing the hematite ore on a laboratory scale and calculating the efficiency of the system during heating and cooling periods.

## Figures and Tables

**Table 1:** Minerals constituting the samples with their chemical composition and discretion.

Mineral	Chemical Composition	Description	Semi-Quantification
Hematite	Fe${}_{2}$O${}_{3}$	Iron Oxide (with two atoms of Fe)	68%
Quartz	SiO${}_{2}$	Silicate	29%
Muscovite	KAl${}_{2}$(Si${}_{3}$Al)O${}_{10}$(OH,F)${}_{2}$	Phyllosilicate	0.5%
Apatite	Ca${}_{5}$(PO${}_{4}$)${}_{3}$	Calcium phosphate	2%

**Table 2:** XRF analysis of TD-AQ and TD-NAQ lots, illustrating the major and trace elements in wt.% and ppm, respectively.

Elements	TD-AQ	TD-NAQ
	**Major Elements (wt.% )**	
Na${}_{2}$O	0.253	0.274
Al${}_{2}$O${}_{3}$	6.304	6.493
SiO${}_{2}$	23.797	24.206
P${}_{2}$O${}_{5}$	0.623	0.627
K${}_{2}$O	0.989	1.034
CaO	0.124	0.123
TiO${}_{2}$	0.184	0.180
Fe${}_{2}$O${}_{3}$	66.527	65.682
Ce	QHigh	0.029
LOI	0.990	0.960
	**Trace Elements (ppm)**	
Cr	30.7	33.1
Cu	ND	12.3
Rb	42.2	43.4
Y	95.6	96.4
Zr	69.6	61.5
La	41.5	38.0
Ce	94.4	QHigh
Nd	48.2	49.6
W	18.4	35.3
Pb	27.6	29.9

**Table 3:** Thermal and physical properties of TD, OS and US lots.

Parameters	Thermal Conductivity (W/mK)	Specific Heat (J/kgK)	Density (kg/m${}^{3}$)	RMS Error
TD	0.31	338	2171	0.42
OS	0.32	321	1926	0.33
US	0.252	316	2262.7	0.23

**Table 4:** Specific heat of each lot corrected for the solid fraction.

Parameters	Porosities	Specific Heat Solid (J/kgK)
TD	0.453	618.13
OS	0.515	661.68
US	0.430	554.44

## Data Availability

Source data are available from the corresponding author upon reasonable request.

## Abbreviations

The following abbreviations are used in this manuscript:

DAQ	Data Acquisition
RMS	Root Mean Square
XRD	X-ray Diffraction
XRF	X-ray Fluorescence
US	Undersize
TD	Total Distribution
OS	Oversize
